# Knowledge, Attitude, and Practices (KAPs) of Community Pharmacists Regarding COVID-19: A Cross-Sectional Survey in 2 Provinces of Pakistan

**DOI:** 10.1017/dmp.2021.54

**Published:** 2021-02-16

**Authors:** Khayal Muhammad, Muhammad Saqlain, Gul Muhammad, Ataullah Hamdard, Muhammad Naveed, Muhammad Hammad Butt, Siraj Khan, Najlaa Saadi Ismael, Zakir Khan, Yusuf Karatas

**Affiliations:** 1 Department of Clinical Pharmacy, Faculty of Pharmacy, Near East University, Nicosia, North Cyprus, Turkey; 2 Department of Pharmacy, Quaid-i-Azam University, Islamabad, Pakistan; 3 Department of Medicine, Hayatabad Medical Complex, Peshawar, Khyber Pakhtunkhwa, Pakistan; 4 Faculty of Sciences and Arts, Department of Statistics, Cukurova University, Adana, Turkey; 5 Department of Pharmacology and Pharmacotherapy, Faculty of Medicine, University of Szeged, Szeged, Hungary; 6 Faculty of Pharmacy, University of Central Punjab, Lahore, Pakistan; 7 Department of Clinical Sciences, Faculty of Pharmacy, Philadelphia University, Amman, Jordan; 8 Çukurova Üniversitesi, Tıp Fakültesi, Tıbbi Farmakoloji Anabilim Dalı, Sarıçam, Adana, Turkey; 9 Balcalı Hospital, Cukurova University, Adana, Turkey

**Keywords:** awareness, community pharmacists, COVID-19, infections, KAPs study

## Abstract

**Objective:**

The 2019 coronavirus disease (COVID-19) is a global pandemic with no therapy, and pharmacists being a part of the health care system have a vital role in the management of COVID-19. The purpose of this study is to assess the knowledge, attitude, and practices of community pharmacists (CPs) regarding COVID-19.

**Method:**

An online survey was conducted among 393 CPs in 2 provinces of Pakistan during the lockdown period. A validated questionnaire (Cronbach’s alpha, 0.745) was used for data collection. All statistical analyses were analyzed by using SPSS, version 21 (IBM Corp, Armonk, NY).

**Results:**

Among 393 participants, 71.5% (n = 281) had good knowledge, 44% (n = 175) had a positive attitude, and 57.3% (n = 225) had good practices regarding COVID-19. Social media (45.29%, n = 178) were reported as the main source to seek information of COVID-19. Good knowledge, age ≥ 26 years, and a PhD degree level were the substantial determinants (*P* = < 0.05) of a good attitude. Similarly, a CP with experience of > 5 years, a PhD degree, good knowledge, and a good attitude has higher odds of having good practices as compared with reference categories (*P* = < 0.05).

**Conclusion:**

In short, a majority of the CPs had good knowledge but had a poor attitude and practice toward the management of COVID-19. Standard-structured educational and counseling programs for CPs regarding COVID-19 are needed for effective management.

## Introduction

The 2019 coronavirus disease (COVID-19), caused by severe acute respiratory syndrome coronavirus 2 (SARS-CoV-2), emerged from Wuhan, China, in December 2019 and created a remarkable panic in China and globally. Cases of COVID-19 were no longer limited to China, with the increasing number of cases and widening geographical spread raising grave concerns about the future trajectory of the outbreak.^1^ Coronavirus is a zoonotic beta-coronavirus similar to the viruses responsible for causing SARS in China and the Middle East respiratory syndrome (MERS) in Saudi Arabia. SARS-CoV-2 is spread through close contact with persons, coughing, and sneezing. Major symptoms are fever, dry cough, shortness of breath, and may sometimes include severe diseases, such as pneumonia, respiratory distress, and death.^2,3^ The World Health Organization (WHO) declared the COVID-19 outbreak a public health emergency and a global pandemic on March 11, 2020.^1^ According to the WHO situation report – 180 on COVID-19, by July 18, 2020, there were 138 76 441 confirmed cases and 593 087 deaths reported globally.^4^

Experience gained from the previous SARS outbreak in 2002 suggests that knowledge and attitude toward contagious infections are linked with a level of panic affection amid the community, which can further complicate chances to inhibit the transmission of the infection.^5^ Therefore, it is imperative that the community be equipped with empirically precise knowledge and tools to address and cope with the impact of COVID-19 effectively. Currently, COVID-19 is a public health emergency and needs cooperation by individuals to halt further transmission by following given guidelines, government orders, and a range of preventive measures.^6^ The health care system of different countries started effective planning to cope with the COVID-19 pandemic. Pharmacists are an important part of the health care system, and their role is critical in completing the management cycle of the new coronavirus outbreak.^3,7^ Pharmacists being a member of the health care team can play an important role in disease management and outbreak surveillance.^8^ Globally, in the current situation of lockdown, pharmacies are among the few shops or places that are kept open for public services, and therefore community pharmacists (CPs) are the first point of contact to the community to help fulfill their health care needs.^9^

The International Pharmaceutical Federation (FIP) emphasized the effective role of pharmacists in the community for preventing the spread of COVID-19.^10^ The CPs often act as a reliable information source for individuals having concerns or needing information and advice regarding ailments. Moreover, pharmacists are readily available at community pharmacies and accessible to the general population.^9^ The CPs across the world have a central role to play an effective role in COVID-19 preventive measures and action.

A study from China acknowledged that community pharmacy management teams shall support different services by providing an adequate supply of COVID-19-related medications and preventive products, in addition to providing sufficient staff training.^11^ From Colombia, CPs were proposed to contribute to the early detection and appropriate referral of possible cases of the virus, then to be reported through designated telephone lines, in addition to providing patient education.^12^ Health care teams include pharmacists who are responsible for providing knowledge, delivering good quality management, and protecting individuals from an illness during the pandemic period. Therefore, pharmacy students’ education should include training in precautionary measures, effective treatment, and follow-up. This is critical, together with their behavior in these fields. Normally, training in disaster medicine occupies a very small place in regular medical curricula globally.^13^

Soon, the confirmed and fatality cases of COVID-19 are expected to increase significantly in limited resources due to the weak health care system and facilities.^14^ Pakistan is a low-middle income and limited resource country with the most vulnerable geographical surroundings as it shares borders with the most affected countries, such as China, India, and Iran. According to the WHO, COVID-19 situation report 180: 261 917 cases and 5522 deaths were reported in Pakistan.^4^ The Pakistani health care system faces a lack of basic health facilities, recommended policies, unavailability of proper medical equipment, financial crisis, and a limited number of health care professionals (HCPs) to handle such an outbreak.^15^ There is limited availability of the CPs in Pakistan, and also their role is not properly defined.^16^ The fight against COVID-19 is continuing in Pakistan, and the involvement of CPs and their role is needed to effectively manage the fight against this global threat.

The knowledge, attitude, and practices (KAPs) survey provides a design to evaluate existing programs and to identify effective strategies for behavior change in society. Currently, there is limited information regarding the awareness level of CPs in Pakistan. The KAPs survey offers a convenient plan to check the existing programs and to find out adequate approaches for behavior change in the community. To promote COVID-19 outbreak management in Pakistan, it is very important to find out the awareness of CPs about COVID-19 in the current serious situation. In the present research, we determined the KAP of CPs toward COVID-19 during the fast rise period of the pandemic.

## Methodology

### Study Design

An online cross-sectional survey was conducted from April 10–30, 2020, during the lockdown period. During this time, it was difficult to conduct a community-based sampling survey; therefore, we preferred using a convenient sampling model for data collection from 2 provinces (Punjab and Khyber Pakhtunkhwa) of Pakistan. Total registered pharmacists in Pakistan are 34 000. From this number, 26 000 of them are registered in Punjab and 3000 in Khyber Pakhtunkhwa.^17^ Lahore and Peshawar are the metropolitan cities of Punjab and Khyber Pakhtunkhwa, respectively, and represent the remaining cities. CPs were sought through different pharmacist groups, academic institutions, colleague pharmacists, and other sources, and the questionnaire was shared with them. This study was carried out among CPs who are educated and trustworthy and responded to the questionnaire with honesty and professionalism. In both cities, a prodigious number of pharmacies are present where CPs serve the health care profession and patients. A study reported that only 10% (n = 2900) of registered pharmacists are working at community pharmacies in Pakistan.^16^

### Sample Size

The minimum obligatory sample size calculated was 340 by using Raosoft software,^18^ with a population size of 2900. Response distribution was assumed as 50%, power was kept at 80%, the margin of error 5%, and a 95% confidence level was chosen for sample estimation. Taking into consideration an additional 20% (n = 68) for nonresponse, inappropriate responses, and error in questionnaire filling, a final sample size of 408 pharmacists will be required.

### Data Collection

The survey was started on April 10, 2020, and response acceptance was closed on April 30, 2020, when the required sample size was achieved. An online questionnaire was sent to the CPs via various social media sources (WhatsApp, Facebook, Gmail, and LinkedIn). Pharmacists working in hospitals, the pharmaceutical industry, and marketing industries were excluded from this study.

### Data Collection Tool

A questionnaire was developed according to the COVID-19 guidelines of the FIP for pharmacists and the pharmacy workforce,^10^ and the national action plan for COVID-19 in Pakistan.^19^ A pilot study was conducted on 40 pharmacists, 20 from each province. The reliability coefficient was estimated by using SPSS, version 21 (IBM Corp, Armonk, NY), and Cronbach’s alpha was 0.745.

There were 5 sections of the questionnaire: demographics, basic questions, and KAPs. Demographic variables included age, gender, work areas, marital status, pharmacy ownership, CP experience, and qualifications. Basic questions contained the source of knowledge, need for more education and training, and the level of concern about COVID-19. The third part of the questionnaire was related to knowledge, and this section had 24 questions where 1 point was assigned to the correct answer and 0 points were assigned to the wrong answer. A score of ≤ 19 indicted poor knowledge and a score of ≥ 20 demonstrated good knowledge. The fourth section was on attitude and had 13 questions on a 5-point Likert scale as strongly disagree = 1, disagree = 2, uncertain = 3, agree = 4, and strongly agree = 5. A score of ≤ 52 indicated a poor attitude and a score of ≥ 53 demonstrated a good attitude. The last part of the questionnaire was composed of 8 questions related to the practice, and each question was scored as “yes” (1-point), “no” (0-point), and “sometimes” (0-point). A score of ≤ 6 demonstrated poor practice, whereas a score of 7–8 indicated good practice.

### Ethical Approval

The study was carried out according to the Declaration of Helsinki and conducted in a strict lockdown period when educational institutes were also closed; hence, the study protocol was approved by the ethical committee of the teaching hospital (Reference number: 817/THQ/HR).

### Statistical Analysis

A statistical analysis was performed using SPSS version 21 (IBM Corp, Armonk, NY). Descriptive statistics were measured as frequencies and percentages for categorical variables. Chi-square tests were used to check a difference in KAP by participants’ characteristics. Multivariable binary logistic regression models were employed to find potential factors linked with good KAP. Results were stated as odds ratios accompanied by a 95% confidence interval. Pearson correlation tests were applied to determine the nature of correlation among KAP sections. A *P*-value of less than 0.05 was considered as a level of significance.

## Results

Participants totaling 393 (15 responses were excluded due to errors in completing questionnaires) were included in the final analysis, of which half (52.2%, n = 205) of the respondents were male, 71% (n = 279) were single, and a similar proportion (72.3%, n = 284) was working as an employee in a pharmacy. The majority of the CPs (67.4%, n = 265) had related work experience of 5 years or less, 69% (n = 271) of the participants had a PharmD degree, and 85.8% (n = 337) wished to obtain more education and training regarding COVID-19 (Table 1).

**Table 1.** Demographic characteristics of community pharmacists

Variable	Frequencies	Percentages
**Gender**		
Male	205	52.2
Female	188	47.8
**Age**		
≤ 25 years	124	31.5
≥ 26 years	269	68.5
**Marital status**		
Married	114	29.0
Single	279	71.0
**Pharmacy ownership**		
Owned	67	17.0
Partnership	42	10.7
Employed	284	72.3
**Experience**		
≤ 5 years	265	67.4
> 5 Years	128	32.6
**Education?**		
B-Pharm	6	1.5
Pharm-D	271	69.0
Master/MPhil	107	27.2
PhD	9	2.3
**Need of education?**		
Yes	337	85.8
No	56	14.2
**Level of concern?**		
Very high concern	231	58.8
High concern	122	31.0
Moderate concern	35	8.9
Low concern	3	0.8
Very low concern	1	0.3

**Figure 1.**
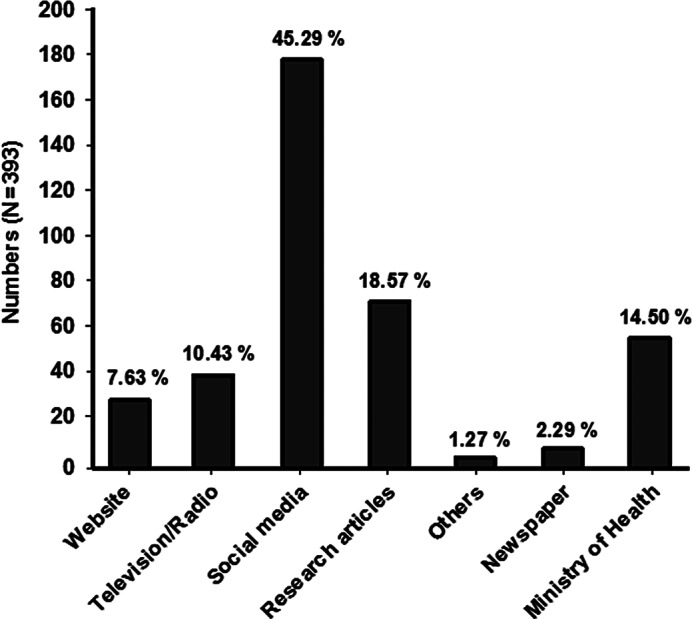
The various information sources used by community pharmacists (*n* = 393).

The majority of the respondents used social media (45.29%, n = 178) as the main source to seek information regarding COVID-19 followed by research articles (18.57%, n = 73) and the ministry of health website (14.5%, n = 57) (Figure 1).

### Knowledge Among CPs Regarding COVID-19

Overall, the majority of respondents (71.5%, n = 281) had good knowledge regarding COVID-19. More than 90% of the participants had good knowledge regarding the nature, symptoms, risky groups, and transmission of COVID-19. Additionally, 81.6% of participants were aware that there is no vaccine available in the market, and 98.2% reported that gathering in crowds is one of the major factors in the spread of disease. Only 30.8% didn’t know that it is a zoonotic disease, and 28.8% reported that antibiotics are the first-line treatment (Supplementary File 1).

### Attitude Among CPs Regarding COVID-19

Attitude-related findings demonstrated that only 44% (n = 173) of the CPs had a positive attitude toward COVID-19. Moreover, 65.4% strongly agreed that COVID-19 is a world health concern, less than half (47.6%) agreed that it is also a problem in Pakistan, 50.6% strongly agreed that pharmacists should avail themselves of updated knowledge, and more than 92% of the respondents strongly agreed that CPs can play an important role in this pandemic. Only 36.1% of the CPs disagreed that the Pakistani populations have sufficient information, and 20.4% disagreed that government institutions can control the pandemic (Supplementary File 2).

### Practice Among CPs Regarding COVID-19

**Figure 2.**
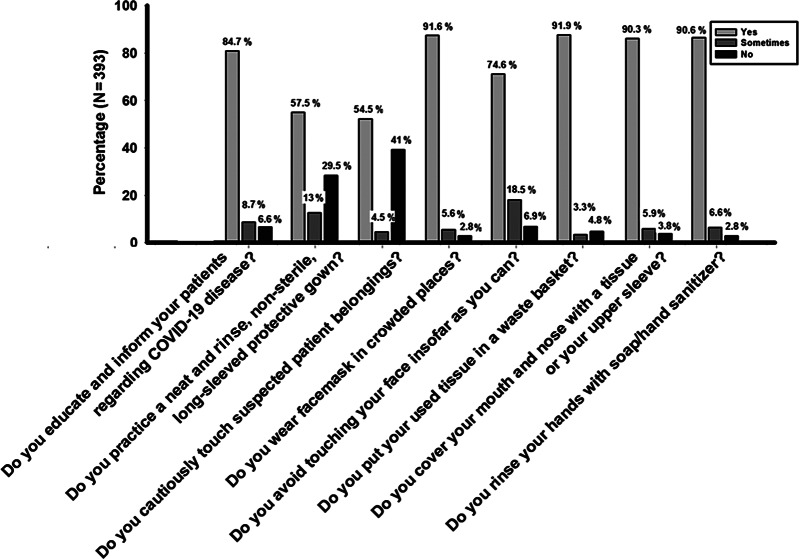
Practices among community pharmacists regarding COVID-19.

More than half (57.3%, n = 225) of the CPs had followed good practices regarding COVID-19. The majority had good practice regarding each item with the highest practice showed in putting used tissue in the basket (91.9%), wearing a face mask (91.6%), washing hands (90.6%), and covering the eyes and nose with tissue (90.3%). A lower percentage of good practice was observed among CPs in wearing protective gowns (57.5%) and in avoiding touching of eyes, nose, or mouth (74.6%) (Figure 2).

### Difference in KAPs Score Among CPs About COVID-19

Chi-square tests were used to assess a difference in KAP by sample characteristics. CP knowledge regarding COVID-19 was significantly different by gender (χ^2^ = 18.78, *P* = < 0.001) and level of concern (χ^2^ = 11.603, *P* = 0.021). The respondents who had a very high concern regarding the COVID-19 pandemic in Pakistan also had good knowledge as compared with those pharmacists who had less concern. Results showed that more than half (56%, n = 220) of the CPs had a poor attitude regarding COVID-19. Factors that differentiated attitude status were gender (χ^2^ = 4.789, *P* = 0.033), education level (χ^2^ = 6.093, *P* = 0.047), and level of concern (χ^2^ = 12.886, *P* = 0.005).

Of the 393 CPs, only 57.3% had good practice in following precautionary measures regarding COVID-19. Findings demonstrated that female pharmacists had good practice in following precautions as compared with male pharmacists (χ^2^ = 5.383, *P* = 0.020). Other factors that significantly associated with practice status were age (χ^2^ = 5.322, *P* = 0.025) and education level (χ^2^ = 9.831, *P* = 0.020) (Table 2).

**Table 2.** Difference in knowledge, attitude, and practice by sample characteristics

Variables	Knowledge			Attitude			Practice		
	Poor	Good	χ^2^ (*P*)	Poor	Good	χ^2^ (*P*)	Poor	Good	χ^2^(*P*)
	(n [%])	(n [%])		(n [%])	(n [%])		(n [%])	(n [%])	
**Total**	112 (28.5)	281 (71.5)		220 (56.0)	173 (44.0)		168 (42.7)	225 (57.3)	
**Gender**			18.78 **(< 0.001)**			4.789 (**0.033**)			5.383 (**0.020**)
Male	39 (19.0)	166 (81.0)		104 (50.7)	101 (49.3)		99 (48.3)	106 (51.7)	
Female	73 (38.8)	115 (61.2)		116 (61.7)	72 (38.3)		69 (36.7)	119 (63.3)	
**Age**			0.775 (0.379)			10.171 (0.001)			0.049 (0.825)
≤ 25 years	39 (31.5)	85 (68.5)		84 (67.7)	40 (32.3)		52 (41.9)	72 (58.1)	
≥ 26 years	73 (27.1)	196 (72.9)		136 (50.6)	133 (49.4)		116 (43.1)	153 (56.9)	
**Marital status**			1.827 (0.172)			0.166 (0.684)			5.322 (**0.025**)
Married	27 (23.7)	87 (76.3)		62 (54.4)	52 (45.6)		59 (51.8)	55 (48.2)	
Single	85 (30.5)	194 (69.5)		158 (56.6)	121 (43.4)		109 (39.1)	170 (60.9)	
**Pharmacy ownership**			0.894 (0.674)			0.832 (0.660)			0.700 (0.705)
Owned	16 (23.9)	51 (76.1)		35 (52.2)	32 (47.8)		26 (38.8)	41 (61.2)	
Partnership	12 (28.6)	30 (71.4)		22 (52.4)	20 (47.6)		17 (40.5)	25 (59.5)	
Employed	84 (29.6)	200 (70.4)		163 (57.4)	121 (42.6)		125 (44.0)	159 (56.0)	
**Experience**			0.013 (0.909)			0.259 (0.611)			2.136 (0.144)
≤ 5 years	76 (28.7)	189 (71.3)		146 (55.1)	119 (44.9)		120 (45.3)	145 (54.7)	
> 5 Years	36 (28.1)	92 (71.9)		74 (57.8)	54 (42.2)		48 (37.5)	80 (62.5)	
**Education level**			1.760 (0.624)			6.093 (**0.047**)			9.831 (**0.020**)
B-Pharm	3 (50.0)	3 (50.0)		5 (83.3)	1 (16.7)		3 (50.0)	3 (50.0)	
Pharm-D	78 (28.8)	193 (71.2)		154 (56.8)	117 (43.2)		107 (39.5)	164 (60.5)	
Master/MPhil	28 (26.2)	79 (73.8)		59 (55.1)	48 (44.9)		57 (53.3)	50 (46.7)	
PhD	3 (33.3)	6 (66.7)		2 (22.2)	7 (77.8)		1 (11.1)	8 (88.9)	
**Need of education?**			0.392 (0.531)			1.828 (0.176)			0.320 (0.572)
Yes	98 (29.1)	239 (70.9)		184 (54.6)	153 (45.4)		146 (43.3)	191 (56.7)	
No	14 (25.0)	42 (75.0)		36 (64.3)	20 (35.7)		22 (39.3)	34 (60.7)	
**Level of concern?**			11.603 **(0.021)**			12.886 **(0.005)**			4.576 (0.334)
Very high concern	68 (29.4)	163 (70.6)		113 (48.9)	118 (51.1)		97 (42.0)	134 (58.0)	
High concern	29 (23.8)	93 (76.2)		80 (65.6)	42 (34.4)		48 (39.3)	74 (60.7)	
Moderate concern	11 (31.4)	24 (68.6)		24 (68.6)	11 (31.4)		19 (54.3)	16 (45.7)	
Low concern	3 (100.0)	0 (0.0)		1 (33.3)	2 (66.7)		2 (66.7)	1 (33.3)	
Very low concern	1(100.0)	(0.0)		1 (100.0)	(0.0)		1 (100.0)	0 (0.0)	

### Factors Associated With Good KAP About COVID-19

A binary logistic regression analysis was performed to evaluate the substantial determinants of good KAP about COVID-19 among CPs in Pakistan. Findings indicated that female participants had 0.243 times lower odds (OR: 0.243, 95% CI: 0.135–0.436, *P* = < 0.001) of good knowledge as compared with male counterparts. Results revealed that age ≥ 26 years (OR: 2.046, 95% CI: 1.214–3.450. *P* = 0.007), PhD degree level (OR: 14.16, 95% CI: 0.875–22.95, *P* = 0.042), and good knowledge (OR: 2.045, 95% CI: 1.233–3.391, *P* = 0.006) were the significant determinants of a good attitude. Similarly, a CP who had experience > 5 years (OR: 1.505, 95% CI: 0.944–2.40, *P* = 0.048), a PhD degree (OR: 6.574, 95% CI: 0.362–11.942, *P* = 0.021), good knowledge (OR: 2.448, 95% CI: 1.485–2.376, *P* = < 0.001), and a good attitude (OR: 1.518, 95% CI: 0.970–2.376, *P* = 0.048) had higher odds of good practice as compared with reference categories (Table 3). The Pearson correlation tests showed a linear statistically significant positive correlation amid KAP scores as follows: knowledge-attitude (r = 0.274, *P* = < 0.001), attitude-practice (r = 0.170, *P* = 0.001), and knowledge-practice (r = 0.341, *P* = < 0.001) (Table 4).

**Table 3.** Regression analysis for factors associated with good knowledge, attitude, and practice regarding COVID-19

Variables	Knowledge		Attitude		Practice	
	OR (95% CI)	*P*	OR (95% CI)	*P*	OR (95% CI)	*P*
**Gender**						
Male	1.00	−	1.00	−	1.00	−
Female	0.243 (0.135-0.436)	**< 0.001**	0.859 (0.507-1.455)	0.572	1.79 (1.038-3.086)	
**Age**						
≤ 25 years	Reference	−	1.00	−	1.00	−
≥ 26 years	1.783 (1.425-2.282)	0.281	2.046 (1.214-3.450)	**0.007**	1.395 (0.822-2.368)	0.217
**Marital status**						
Married	1.00	−	1.00	−	1.00	−
Single	1.022 (0.562-1.857)	0.944	1.181 (0.708-1.969)	0.525	1.607 (0.955-2.704)	0.074
**Pharmacy ownership**						
Owned	1.00	−	1.00	−	1.00	−
Partnership	0.785 (0.180-1.664)	0.991	1.039 (0.440-2.453)	0.931	0.902 (0.379-2.148)	0.816
Employed	0.866 (0.426-1.760)	0.690	1.006 (0.550-1.841)	0.984	0.694 (0.377-1.277)	0.240
**Experience**						
≤ 5 years	1.00	−	1.00	−	1.00	−
> 5 years	1.107 (0.671-1.826)	0.690	1.007 (0.638-1.590)	0.976	1.505 (0.944-2.40)	**0.048**
**Education?**						
B-Pharm	1.00	−	1.00	−	1.00	−
Pharm-D	2.307 (0.207-25.735)	0.390	4.268 (0.440-41.41)	0.211	1.928 (0.163-5.288)	0.133
Master/MPhil	2.707 (0.465-1.748)	0.281	4.663 (0.374-35.83)	0.265	2.618 (1.106-4.621)	0.193
PhD	3.541 (0.623-20.139)	0.497	14.16 (0.875-22.95)	**0.042**	6.574 (0.362-11.942)	**0.021**
**Need of education?**						
Yes	1.00	−	1.00	−	1.00	−
No	1.933 (0.942-3.970)	0.073	0.626 (0.333-1.176)	0.145	1.107 (0.585-2.093)	0.754
**Level of concern?**						
Very high concern	1.00	−	1.00	−	1.00	−
High concern	0.991 (0.496-1.793)	0.220	3.496 (0.308-3.800)	**0.004**		0.491
Moderate concern	0.878 (0.496-1.793)	0.278	3.443 (0.200-3.980)	**0.044**		0.528
Low concern	0.799 (0.013-1.071)	0.999	2.498 (0.198-3.193)	0.479		0.453
Very low concern	0.782 (0.019-1.028)	0.999	2.368 (0.143-3.872)	0.899		0.899
**Knowledge**	−	−				
Poor	−	−	1.00	−	1.00	−
Good	−	−	2.045 (1.233-3.391)	**0.006**	2.448 (1.485-2.376)	**< 0.001**
**Attitude**	−	−	−	−		
Poor	−	−	−	−	1.00	−
Good	−	−	−	−	1.518 (0.970-2.376)	**0.048**

**Table 4.** Correlation between scores of knowledge, attitude, and practice

Variable	Correlation Coefficient	*P*-Value
Knowledge-Attitude	0.274*	**< 0.001**
Attitude-Practice	0.170*	0.001
Knowledge-Practice	0.341*	< 0.001

* Correlation significant at 0.05 level (2-tailed).

## Discussion

Pharmacists play a pivotal role in the provision of drug-related information to patients, caregivers, and health care practitioners.^20^ The FIP emphasizes the active role of pharmacists in the community and hospital setups in preventing the spread of COVID-19.^10^ Pharmacists are easily available and readily accessible to the general population all the time. They are the first point of contact for all those individuals who need medicine and information about medicines.^21-23^ To the best of our knowledge, not a single study assessed KAPs among CPs toward COVID-19 in 2 major provinces of Pakistan.

In this study, it is revealed that the majority of the respondents used social media (45.3%) followed by research articles (18.6%) as their primary source to get information about COVID-19. These results were comparable with the findings of Basheti et al. (59.5%),^3^ Saqlain et al. (87.7%),^24^ and Giao et al. (91.1%).^25^ Conversely, Kara et al. reported that the participants generally used television, newspapers, and the Internet as a principal source to get information about COVID-19.^20^ Moreover, the current study showed that research articles were also searched by CPs as a source of information because valid and satisfactory information can be obtained from standard research articles. The CPs can be misguided by the false information that is available on the Internet and circulates through social media. Therefore, the CPs and other HCPs should carefully evaluate sources of COVID-19 information and use only standard and authentic content to seek information.^20,26^

A good knowledge, positive attitude, and practices among CPs regarding basic precautionary measures (eg, wearing protective clothing, goggles, face mask, and gloves) are important because it decreases the chances of transmission. Moreover, the current pandemic nature of the COVID-19 has made it mandatory for CPs to increase their precautions and follow the recommended hygienic protocols. Good knowledge and practice of CPs in complying with precautionary measures not only create awareness among patients, but also give an important message in society.^3,20^

The majority of respondents (71.5%) in this study had good knowledge regarding COVID-19. Other findings revealed that the knowledge of CPs was significantly different by the level of concern and gender. The recent studies conducted in Turkey (90%) among pharmacists,^20^ in Iran (56.5%) among nurses,^26^ in Pakistan (93.2%),^24^ and in China (88.4%) among HCPs^25^ showed different knowledgeable rates about COVID-19. The results of this study provide confidence in terms of CP knowledge regarding signs, transmission, and preventive measures of COVID-19. Currently, there is no specific antiviral therapy or recommended vaccine for COVID-19, so health practitioners, including CPs, should take all the precautionary steps in the prevention and treatment of this disease.^3,8,24^ Almost 30.80% of the respondents didn’t know it is a zoonotic disease. Similar findings were reported by Kara et al. (30%).^20^ However, it is reported that COVID-19 was closely linked to a wet market in China, and other viral diseases, such as SARS, MERS, Leptospirosis, Crimean-Congo hemorrhagic fever, Dengue, and Ebola emerged from zoonotic origins.^2,3,27-31^ Furthermore, about 28.8% of respondents reported incorrectly that antibiotics are the first-line treatment. At present, COVID-19 treatments include repurposing the available therapeutic drugs.^32^ Antibiotics are not the first-line effective drug in treating COVID-19, and misuse of antibiotics can lead to antimicrobial resistance.^33^

Regression analysis revealed that females had lower odds (OR: 0.243, *P* < 0.001) of good knowledge as compared with their male counterparts (OR: 1.00). However, Zhong et al.^5^ showed that males (vs female, β: -0.284, *P* < 0.001) were significantly associated with lower knowledge scores. Significantly higher knowledge scores among males than females may be related to a higher level of education among males in Pakistan. The finding suggests that intervention via health education can be more efficient if it targets specific demographic groups, such as the COVID-19 knowledge that may be markedly increased if the health education programs are certainly designed for females.^5,24^ The majority of the CPs (92.4%) perceived that they can play an important role in this pandemic. Similar findings were reported by Basheti et al. in Jordan (70%).^3^ Nearly 36.6% of the participants perceived that government health care institutes in Pakistan can control COVID-19, whereas 28.8% disagreed. A recent study conducted in China reported that the majority of the respondents (97.1%) were confident and agreed that their government can win the fight against COVID-19.^5^

The lack of confidence among the participants in the current study may be due to the lack of basic health facilities and recommended policies, absence of proper medical equipment, financial crisis, and weak economic condition of Pakistan to cope with such outbreaks.^15^ Additionally, about 36.10% of the respondents disagreed that the Pakistani populations have sufficient information. This may be due to the perception that the Pakistani population has limited access to the Internet, limited health information resources, and mostly live in rural areas.

This study demonstrated that only 44% of the participants had a positive attitude toward COVID-19. The attitude status of CPs about COVID-19 needs further improvement. These results can be used by the general public, policy-makers, and health workers to analyze the targeted population for the prevention of COVID-19. Regression analysis revealed that CPs of ages ≥ 26 years had higher odds (OR: 2.406, *P* = < 0.001) of demonstrating a good attitude, which is in line with a Turkish study that also reported that age was a factor that influenced participants’ attitudes toward COVID-19 infection.^20^ On the contrary, a study conducted in China did not reveal any association to age, gender, and experience with attitude level.^25^ This variation could be possibly explained by the geographical difference between these countries. Findings also showed that pharmacists with a higher-level degree (PhD) had better (OR: 14.16, *P* = 0.048) attitudes as compared with those with other educational levels. These findings are also supported by Naser et al.^34^ and Zhong et al.^5^ A higher educational degree appreciably increases knowledge and, ultimately, a positive attitude. Targeted educational programs are needed to raise awareness about COVID-19. Such programs need to be tailored to young individuals and particularly to those with lower education levels.^34,35^ A participant who had a higher level of concern demonstrated a good attitude as compared with less concerned pharmacists. The good knowledge among participants was found to be a potential predictor of a positive attitude (OR: 2.045, *P* = 0.006), as knowledge is positively correlated with attitude (r = 0.274, *P* = < 0.001). Our finding was supported by the studies conducted in China,^5^ Iran,^26^ and Thailand.^36^ Therefore, continuous educational programs and periodic training may be an effective intervention to improve the knowledge, level of concern, and, ultimately, attitude of the CP about COVID-19.

More than half (57.3%) of the respondents showed good practices toward COVID-19. The overall highest practice observed among participants was regarding throwing the used tissue in the trash (91.9%) followed by wearing a face mask (91.6%) and washing hands (90.6%). A recently conducted study showed that only 8.9% of the pharmacists used face masks and 84.8% washed their hands at the workplace.^20^ The spread of COVID-19 can be prevented if health care workers wash their hands with soap and water at specific times and maintain an excellent hygiene condition. However, the least practice observed among participants was wearing protective gowns (57.5%). This result is of special concern because good knowledge with poor practice not only increases the transmission of infection but also increases the morbidity and mortality rates in the community.^37^ It was noted that personal protective equipment decreases the transmission of microbes in the hospital setup and also protects people from infections in the community. Therefore, it is mandatory for various CPs and other HCPs to follow the practice guidelines recommended by the National Institute of Health, Islamabad, Pakistan, Centers for Disease Control and Prevention, and the WHO regarding COVID-19. It further suggests that CPs and other health care workers boost their knowledge and attitude that will ultimately translate into good practices.

Findings showed that CPs with good knowledge (OR: 2.44, *P* < 0.01) and a positive attitude (OR: 1.15, *P* = 0.048) demonstrated good practices in following precautionary measures. The studies conducted by Kara et al.,^20^ Saqlain et al.,^24^ and Naser et al.^34^ indicated that pharmacists with good knowledge had a good attitude and showed good practices. Therefore, adequate knowledge is important and could be improved via an extensive educational program for better understanding and improved practices.^38^

### Strengths of the Study

The study respondents were CPs, who are educated and professionals who answered with responsibility. This study was a computer-generated survey and is free of errors as compared to hand-filled proforma. Moreover, it describes CP KAPs toward COVID-19 in detail, which suggests that the health ministry focuses on it. The WHO published materials that were used in the development of the questionnaire, and a 2-step data validation technique was used, hence all these factors increase the reliability of the questionnaire.

### Limitations of the Study

This study was carried out in 2 provinces, and the remaining provinces of Pakistan were not included, hence the study can’t be generalized for the whole country. This study is the unstandardized and inadequate assessment of attitudes and practices toward COVID-19, which should be established through focus group discussion and comprehensive interviews and constructed as multidimensional measures.

## Conclusion

The CPs had good knowledge but had a poor attitude and poor practices toward COVID-19. The majority of the CPs perceived that they can play an important role in this pandemic. This study also highlighted the disparity in some aspects of KAP that must be addressed in future educational, awareness, and counseling programs. It is important for pharmacists to have standard authentic information about COVID-19 and to further convey this knowledge and belief to the community. Future studies are required to evaluate KAPs of other HCPs and other segments of society. This study recommends that the health ministry and other associated authorities promote awareness about COVID-19 and its related symptoms with a comprehensive training program. These programs should be consisting of better-structured targeting not only for medical doctors, but also for pharmacists, nurses, and other paramedical staff to build equilibrium in clinical knowledge about COVID-19.

## Supplementary material

For supplementary material accompanying this paper visit http://dx.doi.org/10.1017/dmp.2021.54. click here to view supplementary material
