# Supplementary material for: Knowledge, Attitude, and Practices (KAPs) of Community Pharmacists Regarding COVID-19: A Cross-Sectional Survey in 2 Provinces of Pakistan
Source: Disaster Med Public Health Prep. 2021 Feb 16:1–9. doi: 10.1017/dmp.2021.54 (PMC8129683; doi:10.1017/dmp.2021.54)
Supplement: Supplementary file 1 [file dmpsup.zip › S1935789321000549sup002.docx]

**Supplementary files**

**Supplementary file 1:** Knowledge among Community pharmacists regarding COVID-19
